# Supplementary figures and images for: Spatio-temporal based deep learning for rapid detection and identification of bacterial colonies through lens-free microscopy time-lapses
Source: PLOS Digit Health. 2022 Oct 5;1(10):e0000122. doi: 10.1371/journal.pdig.0000122 (PMC9931332; doi:10.1371/journal.pdig.0000122)

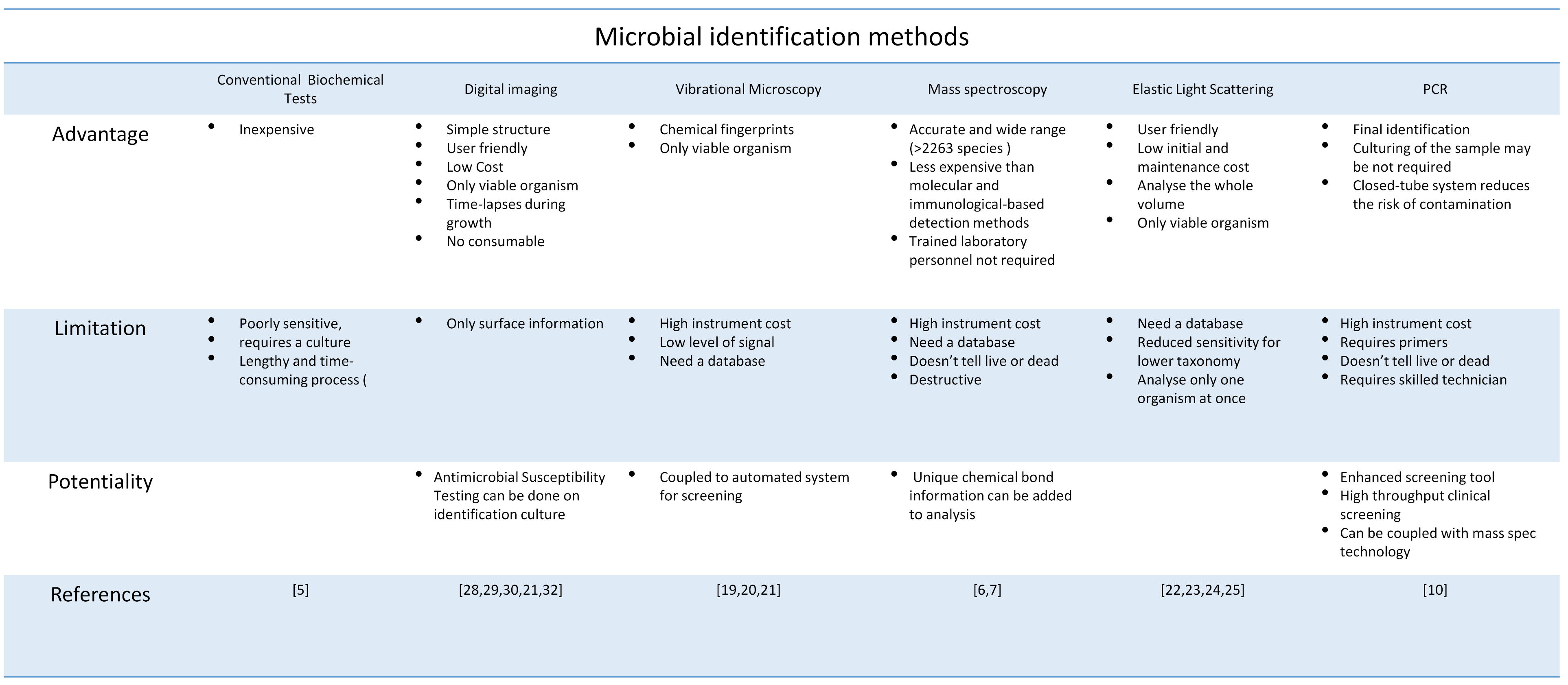

Supplement: S1 Table — Pros and cons of techniques used for bacterial identification. (TIFF) [file pdig.0000122.s001.tiff]

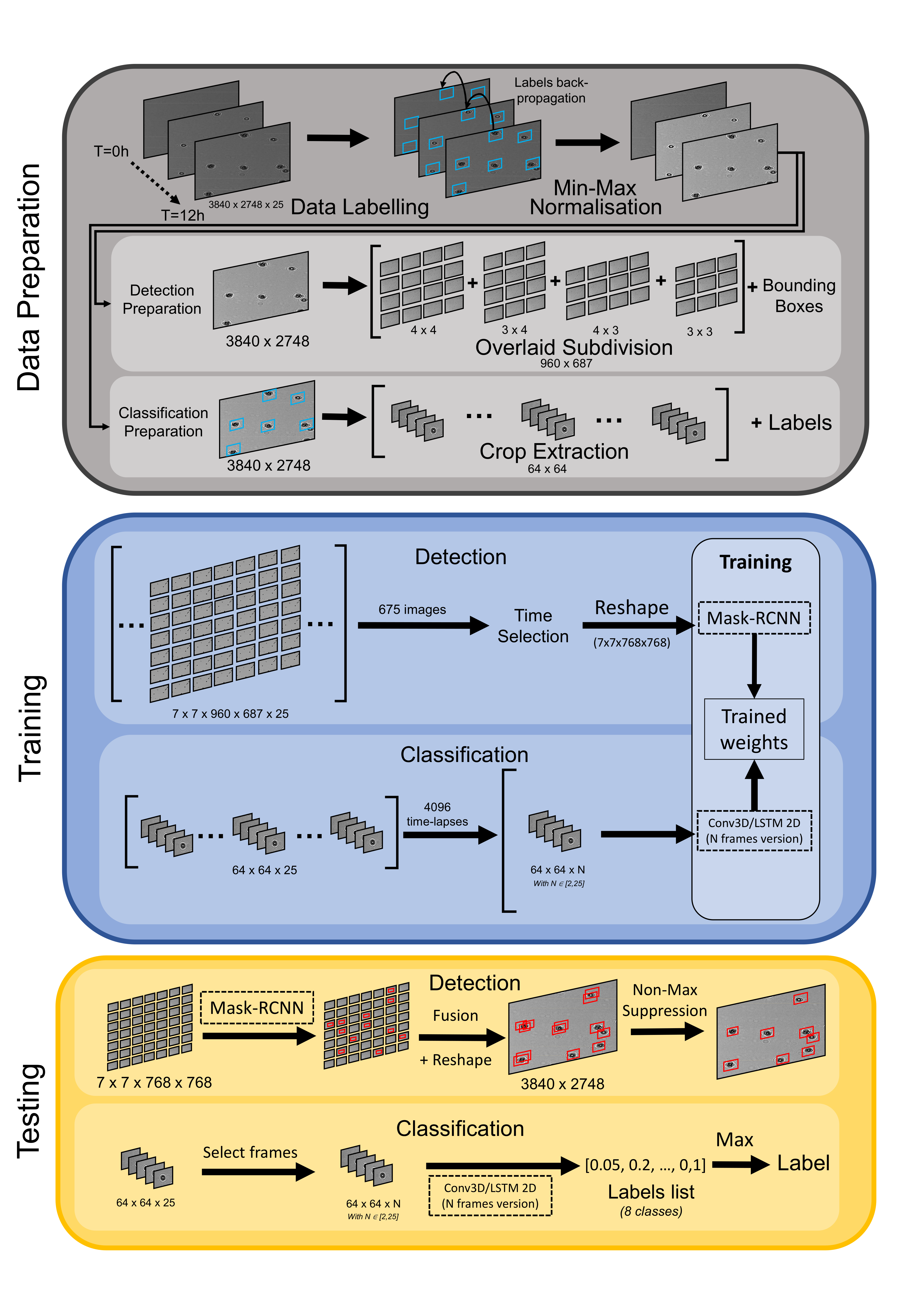

Supplement: S1 Fig — Detailed workflow split in three main phases (Data preparation, Training and Testing) for both detection and classification tasks. (TIFF) [file pdig.0000122.s002.tiff]

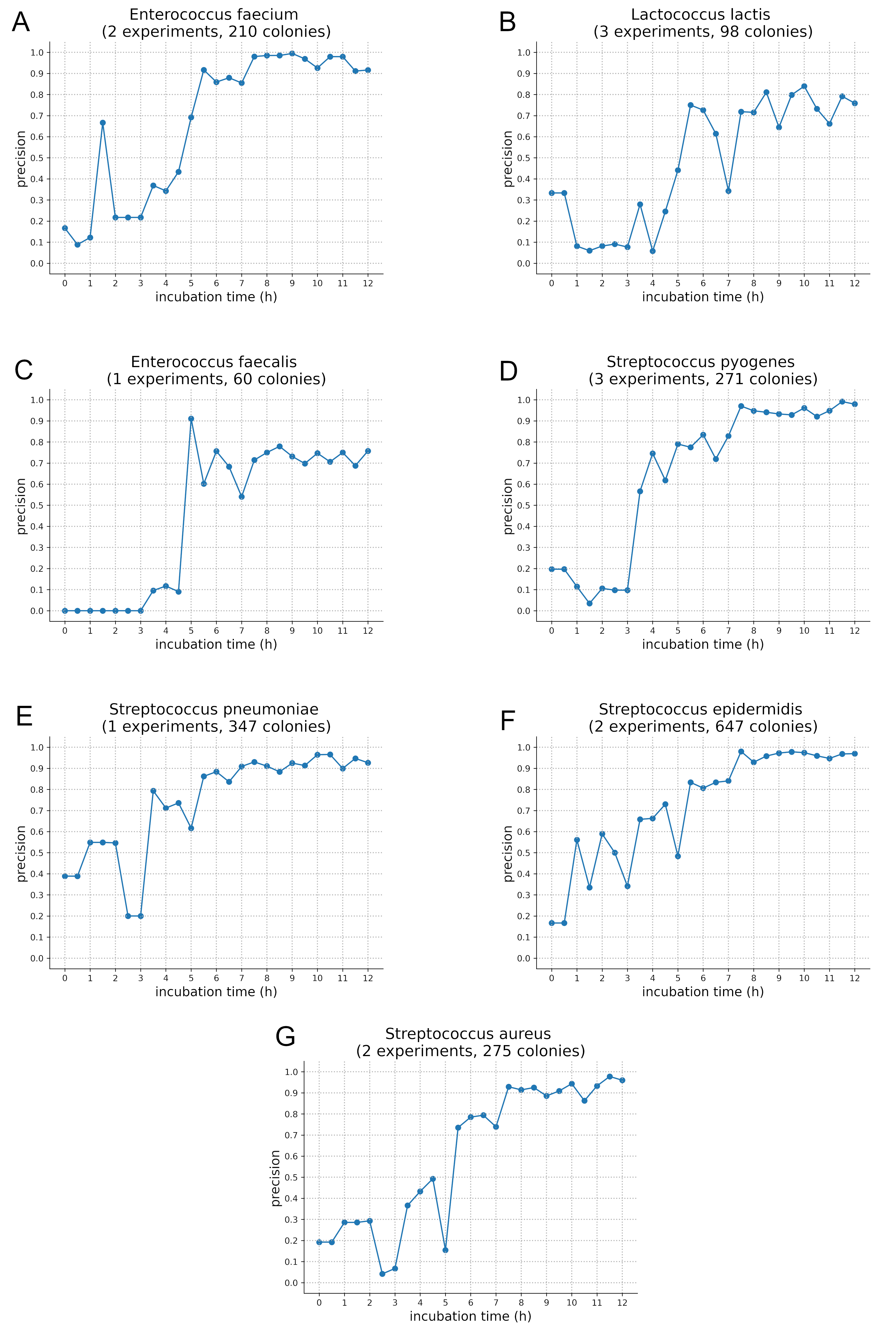

Supplement: S2 Fig — Precision performance during the classification phase over a 12-hour incubation period for (A) E. faecium, (B) L. lactis, (C) E. faecalis, (D) S. Pyogenes (E) S. pneumoniae, (F) S. epidermidis and on (G) S. aureus. (TIFF) [file pdig.0000122.s003.tiff]

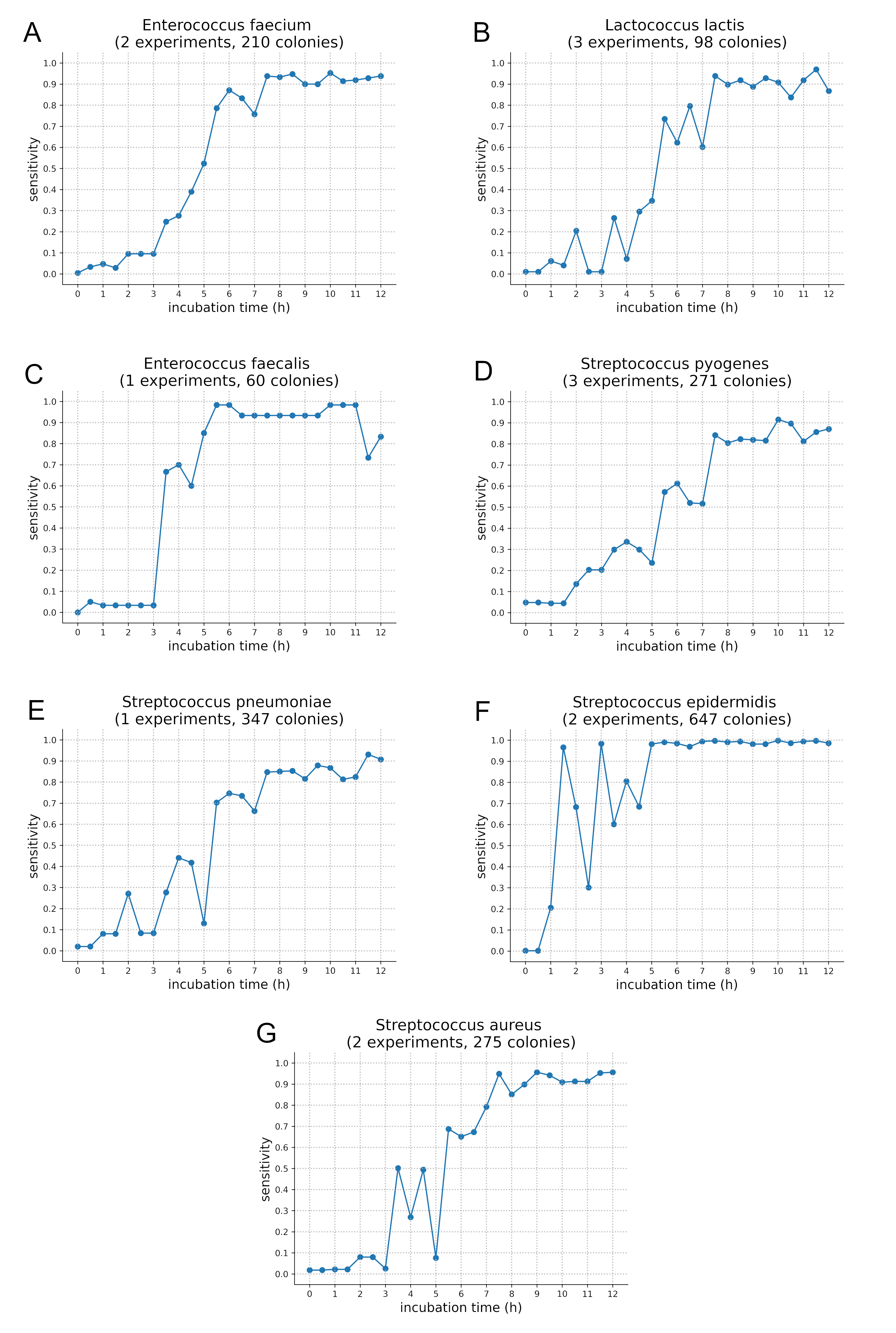

Supplement: S3 Fig — Sensitivity performance during the classification phase over a 12-hour incubation period for (A) E. faecium, (B) L. lactis, (C) E. faecalis, (D) S. Pyogenes (E) S. pneumoniae, (F) S. epidermidis and on (G) S. aureus. (TIFF) [file pdig.0000122.s004.tiff]
