# Supplementary material for: Spatio-temporal based deep learning for rapid detection and identification of bacterial colonies through lens-free microscopy time-lapses
Source: PLOS Digit Health. 2022 Oct 5;1(10):e0000122. doi: 10.1371/journal.pdig.0000122 (PMC9931332; doi:10.1371/journal.pdig.0000122)
Supplement: Attachment — Submitted filename: Article Review-PDIG-D-21-00104.docx [file pdig.0000122.s005.docx]

**Spatio-temporal based deep learning for rapid detection and identification of bacterial**

**colonies through lens-free microscopy time-lapses**- **Review**

A. **SUMMARY OF THE RESEARCH FINDINGS AND OVERALL IMPRESSION**
i. **Summary of the Research findings**

**Abstract section**

The idea of detection and identification of pathogens has been well introduced and the challenge of analyzing complex and large samples has been addressed. An account of the current solutions has been provided as well as the challenges with them. An interesting idea of lens-free imaging combined with deep learning has been proposed to have accurate and quick detection and identification. A good account of results has been provided with good detection rates. However, for each of the detection rates, the authors do not mention how many pathogens were identified. It is therefore important to mention both the detection rate as well as the number of pathogens identified. There is also a statement mentioning the early detection, it is not clearly indicated amongst the problems at the beginning of the abstract that there is the problem of early detection. Its only mentioned that detection is a problem. For this case, the authors provided the time it takes to detect (12hr) but we do not see a comparison with the existing approaches concerning how much time they take. This is critical. Generally, the results see to be good but there is no comparison with what has been done before, so readers cannot confirm the goodness of the results.

**Other sections**

The approach is interesting and mostly I like the part of using un-reconstructed holographic time-lapses which makes the detection and identification faster. This such statement, needs to be captured in the abstract as it is one of the innovativeness of the approach.

It would however be interesting to argue out the issue of holographic reconstruction and no-reconstruction. If previous researchers used reconstruction, why did the current authors choose to use non-reconstruction. Justification is crucial at this point

At some point under the image pre-processing, the authors use the Microsoft COCO format, it would also be important to provide justification for this choice, why not other formats?

There is a grammatical error … line 230/231 (This filter consists in taking highest confidence score predictions and comparing their IOU (intersection over union) with other predictions.

The authors identify one the critical challenges of their approach as indicated in “One major problem is that this architecture is very size-dependant and kernel size cannot be the same for every network” and then make a recommendation of the structure of the classification network to be dynamic depending on the size. This is okay. However, dynamic convolution networks have been mostly used to provide support where there is re-construction yet the authors approach clearly mentions that there is no re-construction to improve detection and identification speed. There is need for clarity on this aspect under section 4.5.2.

The results are impressive, however, it would be good to have a table with comparisons of the authors results and other results in literature with different approaches. This provides clear strength of their work in comparison to others.

It is not clear if the authors if the authors have tested their approach on existing data (if there is any) with similar attributes to have a good comparison with existing research. The fresh data collected may bias the results. Otherwise, the results may make more impact when a test is done on some existing data sets.

ii. **Overall impression.**

This research work is quite impressive, based on the results and the approach taken to attain such good output. There is good hope that this work can be extended to even achieve better identification and detection rates.

B. **SPECIFIC AREAS OF IMPROVEMENT**

- Please include the missing (critical) information in the abstract summary
- Provide a justification on why you choose to use holographic non-reconstruction
- Provide a justification for choosing Microsoft COCO format and not others
- Correct the grammatical errors
- Critically clarify on the issue of using a classification network that is dynamic with respect to holographic non-reconstruction.
- Provide a comparison table with other researchers approaches including their time taken to identify, the detection rates, and possibly which kind of data sets they were dealing with (size is important, because its difficult to compare with work which has bigger data sets with small data sets; definitely there is a big difference in results.
