# Supplementary material for: Spatio-temporal based deep learning for rapid detection and identification of bacterial colonies through lens-free microscopy time-lapses
Source: PLOS Digit Health. 2022 Oct 5;1(10):e0000122. doi: 10.1371/journal.pdig.0000122 (PMC9931332; doi:10.1371/journal.pdig.0000122)
Supplement: Attachment — Submitted filename: Response to Reviewers.docx [file pdig.0000122.s006.docx]

First, we would like to thank the reviewers for their insightful and fruitful comments on our manuscript. It showed us which improvements were necessary and we carefully revised our paper to take into account all their questions and remarks.

In the following letter, we listed the changes we made according to the reviewers’ requests.

**Reviewer #1:**

1.“ It would be interesting to have a better contextualization of your work based on a wider discussion (more than just the six lines (from 100 to106) about other optical (spatial-spectral) bacterial identification methods based on “colony fingerprints”, including for example 1) a wider discussion about forward light scattering research in the same application domain, also considering the contributions of other groups, like <https://doi.org/10.1371/journal.pone.0105272> and <https://doi.org/10.1016/j.bios.2015.01.047>, and 2) other kind of spectral fingerprinting for the same identification aim obtained by hyperspectral imaging, as e.g. <https://doi.org/10.1117/12.2306256> and <https://doi.org/10.1016/j.compbiomed.2017.06.018>. In particular, despite the proposed lens-free approach seems particularly promising for early species identification, also the above methods has been proposed targeting at detection/identification of pathogens in earlier growing stages with respect to conventional reading times. A more informative comparison of the various optical methods that can lead to bacterial identification could be a nice contribution. This could be based on a table with opportunities (pros) and limitations (cons) taking into account several desiderata, like turnaround time, automation, species discrimination power and related species and sub-species differentiation power, consumable constraints (agar type, growing media…)”

We took into consideration this remark from the reviewer and we restructured our small discussion on optical identification methods using as a reference framework the extensive work of Cushla McGoverin et al. “*Optical methods for bacterial detection and characterization*” (2021, <https://doi.org/10.1063/5.0057787>) especially its “Table II” comparing imaging methods based on the optical properties they use and the bacterial properties they highlight as well as their eventual applications. Thus, our new discussion from line 102 to line 122 put in contrast different optical techniques based on what kind of information they try to extract from studied bacteria (biochemical structures, colonies shapes, etc.).

We also merged and updated two identification methods tables from different papers, one by Bae et al. ([10.1586/14787210.2016.1122524](https://doi.org/10.1586/14787210.2016.1122524)) centred around methods studying colony morphologies and the other by Singhai et al.. (<https://doi.org/10.3389/fmicb.2015.00791>) listing several methods used in clinical microbiology. This table is present in the supplementary material.

2. “The differentiation of the proposed method with respect to others already exploiting lens-free imaging is not clear since it is summarized in only 3 lines (from 117 to 119). This is not enough informative and not sufficient to assess the novelty of the work.”

We have taken into account this remark from the reviewer and we have added more details about the specificities of our method compared to other lens-free techniques in competition with ours (please refer to lines 135 to 156 in the new version of the manuscript).

We highlighted the particularities of our thin agar layer culture method allowing a better resolution compared to Petri dish cultures for an identical optical setup, our unique database containing pathogenic bacteria of great interest for further application in the clinical field. We also stressed our use of convolutional networks combined with recurrent networks to allow detection and identification of the strain by extracting spatio-temporal data. In addition, we highlighted the fact that our non-reconstructed holographic approach achieves equivalent scores to other similar studies.

3. “It is not clear whether the cultured bacteria come from ATCC strains or from clinical samples (the fact that the cultures are called “monomicrobial” could lead to think the first or a secondary culture after a first isolation). I think this is a relevant information and, in any cases, some discussion about possible changes in holographic patterns caused by serovars or subspecies should be given.”

We agree with the reviewer that we were unclear about this, changes have been made to explain precisely where our strains are from (lines 159 to 166). Our *Streptococcus pneumoniae* and *Enterococcus faecalis* come from ATCC strains whereas all other strains are clinical isolates. In particular, our Enterecoccus faecium D344 strain comes from the same clinal isolate which was used by Williamson R, et al. In the paper “One or two low affinity penicillin-binding proteins may be responsible for the range of susceptibility of Enterococcus faecium to benzylpenicillin” (1985.) (<https://doi.org/10.1099/00221287-131-8-1933>)

4. “The dataset is limited in terms of number of pathogens and number of colonies. From this point of view this is only a preliminary work. The potential merit of the study to show a possible promising research path for rapid bacteria identification. However, the fact that authors decided to measure results on only 4 bacteria over 7 used for training is unconventional and objectionable. Results should include all 7 species with possible due considerations (as the ones given by the authors) about the fact that only 4 can be considered enough independent/reliable for testing, possibly proposing some results only for a subset related to truly independent datasets. I think that completely avoiding showing results for all the 7 species is not acceptable.”

We fully agree with the reviewer that this study is still a preliminary work. That’s why, to prove our neural networks potential on pathogenic bacteria, we first chose to test them on totally disjointed data (cultures made on different days for the same species, on different sensors, etc.) which forced us to narrow our focus on four of the seven strains used for training. However, we understand that not having the seven species present in the test dataset affect the perception of our work. In consequence, we added new data to the test dataset and reworked some part of our train dataset. Our new datasets are not as strictly disjointed as before as we allowed experiments in the test dataset which were previously put aside due to our restrictive criteria. Nevertheless, no sample were used in both training and testing processes. We now have 26 experiments (4053 colonies) in the train dataset and 13 experiments (1902 colonies) in the test dataset which both contains the seven pathogenic strains. For both train and test dataset we added 72 different background time-lapses which either contain non-bacterial immovable objects (dust or air bubbles) or nothing of interest.

5. “The description given from line 228 to 234 is unclear. A figure could help supporting reader understanding. A figure describing the whole processing workflow lens-free image are subject to should be provided (what happens to images, how images are handled, cropped, sampled…. How images and subimages flow into the networks) to support description given in sec. 4.5 and to add more info since fig.5 is not enough informative.”

In response to this remark, a revision of Figure 4 has been made to address the lack of clarity in our explanations. The workflow is now divided into three main phases and two sub-sections (detection and classification). This should make it easier to understand the whole process and the manipulations done to the images. This new figure being very large it has been moved to the supplementary section.

6. “A general introduction to section 5 before starting with specialized sections is advisable.”

Following this comment, we wrote an introduction to our result section to set the context by discussing our test dataset and the inferences made to characterise each network performance (please refer to lines 369 to 384). We have also warned the reader about the influence of the data on our results. In particular, we have highlighted the link between data quantity and network output quality. We have pointed out that data-dense classes are thus better handled by the networks and are generally less sensitive to performance variations.

7. “What said from line 314 to 317 is not described in the methods. Is performance step up described at lines 340-343 due to this delta-growing verification mechanism? Is it possible consolidate early detection performance (T=4h) looking back from the future times. Moreover, I’m not sure to understand why, since you are interested to the grow dynamic (time-lapse) of the same colony in a certain time interval, you do not follow back the same colony starting from the max time (when detection performance are better) toward the min time. The same concerns apply to why results are divided in time steps in sec. 5.2.2. and fig.8,9,10. It is not reasonable that the same colony over time changed species, therefore I would only expect a unique consensus result for each colony.”

This remark suggests that we were not clear enough about those points and need to explain them in detail. The manuscript has been changed in consequence and we propose to add the supplementary figure S1 describing detection and classification workflows.

We use two different networks for detection and classification. Under real conditions, the second network would use the output of the first network as input, which is not the case in our tests. The test datasets for detection and classification were done in advance and were calibrated so we could evaluate each network performance independently. The detection network is judged by its capacity to detect every colony in the field of view while the classification network is evaluated through its ability to distinguish those colonies. The detection network is applied on one frame only (the last one acquired), while the classification network is applied on a stack of frames starting from the beginning of the recording.

We divide our results in time steps so we can track the evolution of each network performance as if it were being used while the recording was done. In this configuration, we want to apply our classification network at each time mark to see how adding one more image affects the output. This evolution is not necessarily a linear growth as two species could potentially be very different at a certain time and tend to look more alike at time passes by. The output will differ depending on which part of the time-lapse the network is focusing. Alongside other factors, this phenomenon explains why one colony can be labelled as being a certain species at a time mark and another one just after. Our guess is that this variability will certainly decrease as we acquire further data to train our networks.

8. “Claim at lines 410-413 about the possibility of a two-step identification (first genus and then species) is not supported by methodological specifications. Please specify how this two-step decision is or could be made.”

Following this comment, we have added some details on how to do this two-step identification (lines 487 – 491). It is possible to use COCO annotations category and supercategory labels to produce classification at both species and genus level for instance. Otherwise, one could create a custom cost function for a specific triple output containing more information such as Gram, species and strain predictions for example.

Another way would be to implement a class hierarchy as done in W.Goo et al. paper “Taxonomy-Regularized Semantic Deep Convolutional Neural Networks” (2016) to learn discriminative subcategory features as different as possible from grouped supercategory features to differentiate between similar subcategories.

9. “Overall, I do not find the use of deep learning solutions highly justified against more traditional image analysis or machine learning approaches. A deeper discussion on this is necessary along with clear indication about how authors prevent overfitting.”

In our case, deep learning seems to be most relevant as it is fully suited to such complex situations where it is necessary to extract and learn many features in order to give an adequate result, especially on time-lapses where . This is particularly important in the context of lens-free microscopy without reconstruction, as there is no consensus on the features needed for good classification. Deep neural networks can work around this problem by learning discriminative features, thus they do not need any expert-based a priori rules to accomplish their tasks. Moreover, the simplicity of an end-to-end system that can be easily re-trained and whose quality increases with larger dataset seems very promising for the development of a functional tool in real conditions.

Overfitting can be prevented by adding additional data (experimental or synthetic with data augmentation) and implementing a regularized training using a larger validation dataset (for example we split our train dataset between training and validation in a 80/20 fashion but it could be 70/30 or even 60/40) and a dynamic learning rate as we did to avoid local minimums. Overall, overfitting was not a major concern in our study as we had enough high-quality data to train correctly our networks and generalized them enough so they could handle unseen data. We even did some experiments on Listeria monocytogenes samples and had quite satisfying results for our detection network (around 90% detection at 10h) and coherent outputs for our classification network with mainly Streptococcus Aureus or Streptococcus Epidermidis labels (both being Gram positive bacteria like L. monocytogenes). However, we did not include those tests in our paper as they were purely qualitative and no L.monocytogenes were present in any of our dataset.

10. “A thoughtful discussion of results (in a dedicated section again taking into account the literature) is lacking and should be added.”

We agree with the reviewer that comparing our results to the literature would benefit our work. It could show our ability to provide rapid and accurate identifications with scores on par with some state-of-the-art methods. That is why we added more references to our results section to evaluate our outputs (see for example from lines 464 to 468).

11. “Quality (resolution) of figures is low and this impairs understanding especially of fig. 4 and 10.”

We understand that bad resolution might impair full comprehension of our work. That is why, figures have been resized and are now larger and better resolved, some ranging from 300 to 400 dpi.

**Reviewer #2:**

***Abstract section***

1. “The idea of detection and identification of pathogens has been well introduced and the challenge of analysing complex and large samples has been addressed. An account of the current solutions has been provided as well as the challenges with them. An interesting idea of lens-free imaging combined with deep learning has been proposed to have accurate and quick detection and identification. A good account of results has been provided with good detection rates. However, for each of the detection rates, the authors do not mention how many pathogens were identified. It is therefore important to mention both the detection rate as well as the number of pathogens identified.”

We agree with the reviewer that this information might be of interest for the reader, thus we added the number of pathogens identified for each species and our detection rates at T=8h in the abstract.

2. “There is also a statement mentioning the early detection, it is not clearly indicated amongst the problems at the beginning of the abstract that there is the problem of early detection. Its only mentioned that detection is a problem. For this case, the authors provided the time it takes to detect (12hr) but we do not see a comparison with the existing approaches concerning how much time they take. This is critical. Generally, the results see to be good but there is no comparison with what has been done before, so readers cannot confirm the goodness of the results”

We emphasised how important the goal of an early and effective detection and classification was in our approach. In fact, a shorter detection time and a more accurate classification would enable quicker and better suited medication for the patient, limiting the development of bacterial resistance. We also updated our results by pointing out that we achieved significant identification starting from 8 h (17 frames) onwards, not just at T=12h.

***Other sections***

3. “The approach is interesting and mostly I like the part of using un-reconstructed holographic time-lapses which makes the detection and identification faster. This such statement, needs to be captured in the abstract as it is one of the innovativeness of the approach.

It would however be interesting to argue out the issue of holographic reconstruction and no-reconstruction. If previous researchers used reconstruction, why did the current authors choose to use non-reconstruction. Justification is crucial at this point”

We have further developed the positive aspects of unconstructed holographic images (for example, see line 135 and the following). Especially how it fits our optical and biological setup with low density and a sample sensor distance around 150 µm. We added this part in our introduction to highlight its interest and how one can avoid problems due to reconstruction. We emphasised the particularity of our non-holographic approach in the abstract too.

5. “At some point under the image pre-processing, the authors use the Microsoft COCO format, it would also be important to provide justification for this choice, why not other formats?”

We might have not stressed this enough but Microsoft COCO format is one of the most popular format for object detection while being easy to use and containing every essential information about the labelled data (data paths, image sizes, labels, bounding boxes and segmentation polygons). We added a justification in the manuscript from lines 270 to line 275. This is not the case for every format, for instance Pascal VOC format do not include masks but only bounding boxes, which hinders segmentation tasks and especially our Mask-RCNN training. For those reasons and the fact that is widely used to benchmark the performance of segmentation and detection models, we chose to adopt this format to annotate our data.

6. “The authors identify one the critical challenges of their approach as indicated in “One major problem is that this architecture is very size-dependant and kernel size cannot be the same for every network” and then make a recommendation of the structure of the classification network to be dynamic depending on the size. This is okay. However, dynamic convolution networks have been mostly used to provide support where there is re-construction yet the authors approach clearly mentions that there is no re-construction to improve detection and identification speed. There is need for clarity on this aspect under section 4.5.2. “

The problem is not exclusive to architectures using re-construction; the challenge here is to propose a classification network deep enough to achieve satisfactory results without creating errors during implementation. The number of layers must be well chosen so as not to create problems when training the model. The number of layers changes depending on the input and it is necessary to make a dynamic architecture with several models adapted to the complexity of the input.

7. “The results are impressive; however, it would be good to have a table with comparisons of the authors results and other results in literature with different approaches. This provides clear strength of their work in comparison to others […] Provide a comparison table with other researchers approaches including their time taken to identify, the detection rates, and possibly which kind of data sets they were dealing with (size is important, because its difficult to compare with work which has bigger data sets with small data sets; definitely there is a big difference in results) ”

We agree that a comparison table is advisable for a better contextualisation of our work, that’s why we added a table comparing bacteria identification techniques especially their advantages and limitations for classification. When information about the dataset used was at our disposal, we explained its particularities in the main manuscript.

8. “It is not clear if the authors have tested their approach on existing data (if there is any) with similar attributes to have a good comparison with existing research. The fresh data collected may bias the results. Otherwise, the results may make more impact when a test is done on some existing data sets.”

To our knowledge there are still no lens-free time-lapse datasets available for benchmarking and especially not on un-reconstructed data. Moreover, our optical setup is quite unique making direct comparison harder. However, we did some tests on totally unseen data (with Listeria monocytogenes samples especially) and achieved coherent results with good detection and poor classification. Considering this situation, we shared some parts of our dataset for other to use in further research (see <https://github.com/paulpaq98/Holographic_Database>).
