# Supplementary material for: Spatio-temporal based deep learning for rapid detection and identification of bacterial colonies through lens-free microscopy time-lapses
Source: PLOS Digit Health. 2022 Oct 5;1(10):e0000122. doi: 10.1371/journal.pdig.0000122 (PMC9931332; doi:10.1371/journal.pdig.0000122)
Supplement: Attachment — Submitted filename: Response to Reviewers.docx [file pdig.0000122.s007.docx]

We would like to thank the reviewers for their insightful comments on our manuscript and we are pleased to see that our previous revisions met their expectations in general. Thanks to those reviews, we were able to improve the quality of this paper.

In the following letter, we listed the changes we made according to this version:

**Reviewers #1 & #2:**

We did further proofreading, following a remark from reviewer #1, and corrected some grammatical errors and typos in our manuscript.

We also extended our github sample dataset (<https://github.com/paulpaq98/Holographic_Database> ) so it can be used as a minimal database with at least one set for training and testing for each species.

We cannot disclose our full dataset right now for legal concerns due to our partnership with another institute to build up this database and due to the CEA data policy.

Despite this, we provided a code snippet showing how our CONV3D + LSTM2D model was built for a seven-frame classification model as an example in our readme. We also reworked the readme to be more useful to someone who would like to reproduce our paper even without the full dataset.

We believe reproducibility can be achieved despite this by pre-training detection and classification models on larger bacterial databases. Re-training those models on the 336 images provided on our github should be sufficient to reach similar results to those in our paper.

We also updated our reference list by adding doi that were missing (reference n°8) and checking that each reference was available.
